# Supplementary material for: Imaging tissues and cells beyond the diffraction limit with structured illumination microscopy and Bayesian image reconstruction
Source: Gigascience. 2018 Aug 23;8(1):giy126. doi: 10.1093/gigascience/giy126 (PMC6325271; doi:10.1093/gigascience/giy126)
Supplement: GIGA-D-18-00276_Revision_1.pdf [file giy126_giga-d-18-00276_revision_1.pdf]

GigaScience

Imaging tissues and cells beyond the diffraction limit with structured illumination microscopy and Bayesian image reconstruction

--Manuscript Draft--

**Manuscript Number:**	GIGA-D-18-00276R1
**Full Title:**	Imaging tissues and cells beyond the diffraction limit with structured illumination microscopy and Bayesian image reconstruction
**Article Type:**	Data Note
**Funding Information:**	National Institute of General Medical Sciences (1R15GM128166-01) — Dr Guy Hagen<br>National Science Foundation (1727033) — Dr Guy Hagen<br>SCIEX (13.183) — Dr Tomáš Lukeš<br>České Vysoké Učení Technické v Praze (SGS18/141/OHK3/2T/13) — Mr Jakub Pospíšil<br>BioFrontiers Institute, University of Colorado Colorado Springs — Dr Guy Hagen
**Abstract:**	Background<br>Structured illumination microscopy (SIM) is a family of methods in optical fluorescence microscopy that can achieve both optical sectioning and super-resolution effects. SIM is a valuable method for high resolution imaging of fixed cells or tissues labeled with conventional fluorophores, as well as for imaging the dynamics of live cells expressing fluorescent protein constructs. In SIM, one acquires a set of images with shifting illumination patterns. This set of images is subsequently treated with image analysis algorithms to produce an image with reduced out-of-focus light (optical sectioning) and/or with improved resolution (super-resolution).<br><br>Findings<br><br>Five complete, freely available SIM datasets are presented including raw and analyzed data. We report methods for image acquisition and analysis using open source software along with examples of the resulting images when processed with different methods. We processed the data using established optical sectioning SIM and super-resolution SIM methods, and with newer Bayesian restoration approaches which we are developing.<br><br>Conclusion<br><br>Various methods for SIM data acquisition and processing are actively being developed, but complete raw data from SIM experiments is not typically published. Publically available, high quality raw data with examples of processed results will aid researchers when developing new methods in SIM. Biologists will also find interest in the high-resolution images of animal tissues and cells we acquired. All of the data was processed with SIMToolbox, an open source and freely available software solution for SIM.
**Corresponding Author:**	Guy Hagen<br><br>UNITED STATES
**Corresponding Author Secondary Information:**	
**Corresponding Author's Institution:**	
**Corresponding Author's Secondary Institution:**	
**First Author:**	Jakub Pospíšil

Powered by Editorial Manager® and ProduXion Manager® from Aries Systems Corporation

**First Author Secondary Information:**	
**Order of Authors:**	Jakub Pospíšil
	Tomáš Lukeš
	Justin Bendesky
	Karel Fliegel
	Kathrin Spendier
	Guy Hagen
**Order of Authors Secondary Information:**	
**Response to Reviewers:**	Dear Editor:<br><br>We would like to resubmit our manuscript entitled “Imaging tissues and cells beyond the diffraction limit with structured illumination microscopy and Bayesian image reconstruction” for consideration in GigaScience as a data note.<br><br>We would like to thank the referees for their very positive reviews of the paper. Reviewer 1 had a minor point of concern:<br><br>“One minor point the authors may want to address is the difference between coherent interference illumination and the incoherent pattern illumination employed in the manuscript. It seems that interference illumination can generate a sin pattern with highest contrast at cutoff frequency while incoherent pattern illumination may suffer from the gradually cutoff of the incoherent OTF.”<br><br>We thank the reviewer for this comment. The reviewer is correct about this, and we have written some comments and added a new reference in the discussion which should clear up this matter. The modified paragraph now reads:<br><br>“There are several other advantages to the use incoherent illumination in SIM, including removing the need for a pupil plane mask to block unwanted diffraction orders. Also, incoherent imaging of a microdisplay for pattern formation means that the pattern spatial frequency in the sample plane does not depend on the wavelength of the light which is used. On the other hand, in incoherent illumination SIM such as we used here, the contrast of the illumination pattern decreases with increasing spatial frequency according to the incoherent optical transfer function [53]. In coherent illumination SIM [8,9,11], the coherent optical transfer function applies [53], and so the pattern contrast does not decrease with increasing spatial frequency. This means that coherent illumination SIM can more efficiently mix high resolution information from outside the frequency limit into the detection passband of the microscope, thereby potentially achieving better resolution than what we achieved in this work. We achieved a lateral resolution enhancement factor of ~1.8 (Fig. 2), whereas a factor of 2.0 is expected for coherent illumination SIM.”<br><br>In addition to this, we have fixed a few typographical errors.<br><br>We hope that the paper will now be acceptable for publication.<br><br>Sincerely,<br><br>Guy M. Hagen
**Additional Information:**	
**Question**	**Response**
Are you submitting this manuscript to a special series or article collection?	No
**Experimental design and statistics**	Yes

Powered by Editorial Manager® and ProduXion Manager® from Aries Systems Corporation

Full details of the experimental design and statistical methods used should be given in the Methods section, as detailed in our Minimum Standards Reporting Checklist. Information essential to interpreting the data presented should be made available in the figure legends.<br><br>Have you included all the information requested in your manuscript?	
**Resources**<br><br>A description of all resources used, including antibodies, cell lines, animals and software tools, with enough information to allow them to be uniquely identified, should be included in the Methods section. Authors are strongly encouraged to cite Research Resource Identifiers (RRIDs) for antibodies, model organisms and tools, where possible.<br><br>Have you included the information requested as detailed in our Minimum Standards Reporting Checklist?	Yes
**Availability of data and materials**<br><br>All datasets and code on which the conclusions of the paper rely must be either included in your submission or deposited in publicly available repositories (where available and ethically appropriate), referencing such data using a unique identifier in the references and in the "Availability of Data and Materials" section of your manuscript.<br><br>Have you have met the above requirement as detailed in our Minimum Standards Reporting Checklist?	No
If not, please give reasons for any omissions below.	we plan to upload the data to GIGA DB when we are contacted by the Gigascience team

Powered by Editorial Manager® and ProduXion Manager® from Aries Systems Corporation

as follow-up to "**Availability of data and materials**<br><br>All datasets and code on which the conclusions of the paper rely must be either included in your submission or deposited in publicly available repositories (where available and ethically appropriate), referencing such data using a unique identifier in the references and in the "Availability of Data and Materials" section of your manuscript.<br><br>Have you have met the above requirement as detailed in our Minimum Standards Reporting Checklist?<br><br>"	

Manuscript

Click here to access/download;Manuscript;Hagen-SIM-resubmission.docx

Click here to view linked References

Imaging tissues and cells beyond the diffraction limit with structured illumination microscopy and Bayesian image reconstruction

Jakub Pospíšil[1], Tomáš Lukeš[1,2], Justin Bendesky[3], Karel Fliegel[1], Kathrin Spendier[3,4], and Guy M. Hagen[3*]

[1]Department of Radioelectronics, Faculty of Electrical Engineering, Czech Technical University in Prague, Technická 2, 16627 Prague 6, Czech Republic.

[2]Laboratory of Nanoscale Biology, École Polytechnique Fédérale de Lausanne, CH-1015 Lausanne, Switzerland.

[3]UCCS center for the Biofrontiers Institute, University of Colorado at Colorado Springs, 1420 Austin Bluffs Parkway, Colorado Springs, Colorado, 80918, USA.

[4]Department of Physics and Energy Science, University of Colorado at Colorado Springs, 1420 Austin Bluffs Parkway, Colorado Springs, Colorado, 80918, USA.

Contact email addresses

Jakub Pospíšil, pospij27@fel.cvut.cz, ORCID: 0000-0003-3615-5752

Tomáš Lukeš lukestom@fel.cvut.cz

Justin Bendesky jbendesk@uccs.edu

Karel Fliegel, fliegek@fel.cvut.cz, ORCID: 0000-0001-5737-6736

Kathrin Spendier, kspendie@uccs.edu

Corresponding author, Guy M. Hagen, ghagen@uccs.edu, ORCID: 0000-0002-4802-9481

Abstract

Background: Structured illumination microscopy (SIM) is a family of methods in optical fluorescence microscopy that can achieve both optical sectioning and super-resolution effects. SIM is a valuable method for high-resolution imaging of fixed cells or tissues labeled with conventional fluorophores, as well as for imaging the dynamics of live cells expressing fluorescent protein constructs. In SIM, one acquires a set of images with shifting illumination patterns. This set of images is subsequently treated

with image analysis algorithms to produce an image with reduced out-of-focus light (optical sectioning) and/or with improved resolution (super-resolution).

Findings: Five complete, freely available SIM datasets are presented including raw and analyzed data. We report methods for image acquisition and analysis using open source software along with examples of the resulting images when processed with different methods. We processed the data using established optical sectioning SIM and super-resolution SIM methods, and with newer Bayesian restoration approaches which we are developing.

Conclusion: Various methods for SIM data acquisition and processing are actively being developed, but complete raw data from SIM experiments is not typically published. Publically available, high quality raw data with examples of processed results will aid researchers when developing new methods in SIM. Biologists will also find interest in the high-resolution images of animal tissues and cells we acquired. All of the data was processed with SIMToolbox, an open source and freely available software solution for SIM.

Keywords: super-resolution microscopy, SIMToolbox, structured illumination microscopy, open-source software, fluorescence, Bayesian methods, LAMP1, live cell imaging.

Data description

Context

Several methods are now available which are able to extend the resolution of fluorescence microscopy beyond the diffraction limit. These methods include photoactivated localization microscopy [1,2] (PALM, FPALM), stochastic optical reconstruction microscopy [3,4] (STORM, dSTORM), super-resolution optical fluctuation imaging [5,6] (SOFI), stimulated emission depletion microscopy [7] (STED), and structured illumination microscopy [8,9] (SIM).

Of these various methods, SIM is usually regarded as the most useful for imaging live cells, and this method has rapidly gained in popularity. Depending on the optical setup and data processing method used, SIM can achieve optical sectioning (OS-SIM) [10], an effect which greatly reduces out-of-focus

light similar to laser scanning confocal fluorescence microscopy. SIM can also be used for imaging beyond the diffraction limit in fluorescence microscopy. Super-resolution SIM (SR-SIM) [8,9], in its most common implementation [11], uses laser illumination to create a high frequency interference fringe pattern (close to or at the resolution limit of the microscope) to illuminate the sample. In such an experiment, image information with details beyond the limit of spatial frequencies accepted by the microscope is aliased into the acquired images. By acquiring multiple images with shifting illumination patterns, a high-resolution image can be reconstructed [8,9]. Two-dimensional SR-SIM enables a twofold resolution improvement in the lateral dimension [8,9,12,13]. If a three-dimensional illumination pattern is used, a twofold resolution improvement can also be realized in the axial direction [11,14,15]. SIM is perhaps the most attractive super-resolution method for imaging live cells because it does not require high illumination powers, can work with most dyes and fluorescent proteins, uses efficient widefield (WF) detection, and can achieve high imaging rates. SIM has been demonstrated in several applications, including 2D [12,13], and 3D imaging [14,16].

As interest in super-resolution imaging has increased, several alternative approaches for SIM have been introduced which use various kinds of patterned illumination [17–21]. For example, in multifocal structured illumination microscopy (MSIM) [17], a 2D array of focused laser spots is scanned across a sample, and subsequent image processing is used to achieve an image with improved resolution. Structured illumination methods have also been combined with light sheet excitation, a method ideal for imaging live cells [22–26].

In addition to new illumination schemes, alternative data processing methods have also been introduced [27–33]. For example, Orieux et al. suggested a 2D method for SIM reconstruction based on Bayesian estimation [28], and our group showed that Bayesian reconstruction methods in SIM have several potential advantages and can achieve a performance comparable to traditional SIM methods [29]. To allow 3D imaging, our group subsequently introduced maximum *a posteriori* probability SIM (MAP-SIM [30]), a method based on reconstruction of the SIM data using a Bayesian framework. Image

restoration approaches are useful when working with low signal levels in SIM [34], and have been recently reviewed [35].

We present complete raw and analyzed SIM data from several different situations in cell biology studies in which we imaged both live and fixed mammalian cells as well as fixed tissues. We used an alternative approach for SIM illumination which has been previously described [30,36,37]. Our system uses either light emitting diode (LED) or laser illumination, and a fast ferroelectric liquid crystal-on-silicon (FLCOS) microdisplay (also known as a spatial light modulator (SLM)) for SIM pattern definition. SLMs have seen use in SIM and related applications when high speed imaging and flexibility in controlling the spatial and temporal properties of the illumination are priorities [12–14,16,25,37–43]. To analyze the data we used OS-SIM, SR-SIM, and MAP-SIM methods. All of the raw and analyzed data are available on GigaDB, and the analysis software (SIMToolbox) is open-source and freely available [36].

Methods

Cell lines and reagents

All cell lines used were maintained in DMEM supplemented with 10% FCS, 100 U/ml penicillin, 100 U/ml streptomycin, and L-glutamate (Invitrogen) at 37 °C and 100% humidity. Cell lines we used for this study included U2-OS (human bone sarcoma, RRID:CVCL_0042), A431 (human skin carcinoma, RRID:CVCL_0037), and Hep-G2 (human liver carcinoma, RRID:CVCL_0027).

Preparation of samples for imaging

(SIM data 1, Fig. 4) U2-OS cells expressing lysosome-associated membrane protein 1 labeled with green fluorescent protein (LAMP1-GFP) were grown in petri dishes with coverslip bottoms (MatTek) for 24 hours, then imaged them in full medium at room temperature. In this experiment, we used microscopy system 1 (Olympus IX71, Table 2).

(SIM data 2, Fig. 5) A431 cells were grown on #1.5H coverslips (Marienfeld) for 48 hours in normal medium. We washed the cells once with phosphate buffered saline (PBS), pH 7.4, and then

treated the cells with 5 μM DiI-C_{16} (Molecular Probes) in PBS at room temperature for 5 minutes. This probe is a lipid modified with a fluorescent dye that inserts into the plasma membrane of live mammalian cells within a few minutes. We then washed the cells twice with PBS, then imaged them on the SIM system in fresh PBS at room temperature using a coverslip chamber (Biotech). In this experiment, we used microscopy system 3 (Leica DMi8, Table 2).

(SIM data 3, Fig. 6) A prepared slide was acquired (AmScope) containing sectioned rabbit testis stained with hematoxylin and eosin (H&E). In this experiment, we used microscopy system 3 (Leica DMi8, Table 2).

(SIM data 4, Fig. 7) Hep-G2 cells expressing Dendra2-histone 4 [44] were grown on #1.5H coverslips for 24 hours, then fixed for 15 minutes at room temperature with 4% paraformaldehyde. We then permeabilized the cells for 5 minutes at room temperature with 0.1% triton-X100, then washed the cells with PBS. We then labeled the actin cytoskeleton of the cells for 1 hour at room temperature with 5 nM Atto 565 phalloidin, followed by washing the cells with PBS. We finally mounted the coverslips on clean slides using mowiol 4-88 (Fluka). In this experiment, we used microscopy system 1 (Olympus IX71, Table 2).

(SIM data 5, Fig. 8) A prepared slide was acquired (Molecular Probes) containing bovine pulmonary endothelial (BPAE) cells stained with Alexa Fluor 488 phalloidin (to label the actin cytoskeleton) and Mitotracker CMXRos (to label mitochondria). In this experiment, we used microscopy system 2 (Olympus IX83, Table 2).

Table 1 summarizes the imaging parameters used for the different samples.

Microscope setup and acquisition

We used three different home-built SIM setups based on the same general design as described previously [30,36,37] (Figure 1). The three SIM systems were based on Olympus IX71, Olympus IX83, and Leica DMi8 microscopes coupled with sCMOS cameras (Andor) under the control of IQ3 software (Andor). The parameters of the different microscope setups are shown in Table 2.

In each microscope setup, the illumination patterns were produced by a high-speed ferroelectric liquid crystal on silicon (FLCOS) microdisplay (SXGA-3DM, Forth Dimension Displays, 13.6 μm pixel pitch). This particular FLCOS microdisplay has been used previously in SIM [14,16,25,29,30,36,37,45–48], and in other optical sectioning systems such as programmable array microscopy (PAM) [38,42,49]. The display was illuminated by a home-built, three channel LED system based on high power LEDs (PT-54 or PT-120 with DK-114N or DK-136M controller, Luminous Devices) with emission maxima at 460 nm, 525 nm, and 623 nm. The output of each LED was filtered with a band pass filter (Chroma), and the three wavelengths were combined with appropriate dichroic mirrors (Chroma). The light was then vertically polarized with a linear polarizer (Edmund Optics). We imaged the microdisplay into the microscope using an external tube lens (Table 2) and polarizing beam splitter cube (Thor Labs). With any of the setups and when using a 100× objective, single microdisplay pixels are imaged into the sample with a nominal size of 136 nm, thus as diffraction-limited spots. This is important for achieving the highest resolution results [37]. More details are available in the supplementary material of [36]. In one experiment (Figure 8) we used a Spectra-X light source (Lumencor).

The microdisplay allows one to create any desired illumination pattern. In our experiments, the illumination masks consisted of line grids of different orientations (0º, 90º, 45º and 135º). The lines were one microdisplay pixel thick (diffraction limited in the sample when using a 100× objective) with a gap of “off” pixels in between. The illumination line grid was shifted by one pixel between each image acquisition to obtain a shifted illumination mask. The shift between each image was constant, and the sum of all illumination masks resulted in homogenous illumination. Our optical setup, in which an incoherently illuminated microdisplay is imaged into the sample with highly corrected microscope optics, results in much more stable SIM illumination parameters compared to conventional SIM in which the illumination pattern is created by laser interference. We use a unique spatial calibration method to determine, with very high accuracy, the position of the patterned illumination in the sample [37]. This is

a spatial domain process and does not rely on fitting of data to a model except for the assumption that the imaging is linear and shift-invariant.

Insert Figure 1

Table 1 Imaging parameters for the SIM datasets

Data	sample	Label (structure)	Pixel size, nm	Illumination	Exposure time, ms	SIM experiment type	SIM pattern # of angles/ phases	Microscope system used
SIM data 1 (Fig. 4)	Live U2-OS cells	LAMP1-GFP (lysosomes and membrane)	65	LED 480 nm	25	2D time lapse	1/11	1
SIM data 2 (Fig. 5)	Live A431 cells	DiI-C16 (membrane)	65	LED 530 nm	100	3D	4/24	3
SIM data 3 (Fig. 6)	Fixed rabbit testis	Hematoxylin and eosin (structural strain)	65	LED 530 nm	200	3D	1/11	3
SIM data 4 (Fig. 7)	Fixed Hep-G2 cells	Dendra2-H4 (nucleus) Atto565-phalloidin (actin)	65	LED 480 nm LED 530 nm	500	3D	4/24	1
SIM data 5 (Fig. 8)	Fixed BPAE cells	AlexaFluor 488 phalloidin (actin) Mitotracker CMXRos (mitochondria)	65	Lumencor spectra-X 470 nm 550 nm	300	2D	1/11	2

Table 2 Parameters of the microscope systems

Setup	Microscope	Objective	sCMOS Camera	Illumination tube lens focal length and part number
1	Olympus IX71	100×/1.4 UPLSAPO	Andor Neo 5.5	180 mm U-TLU
2	Olympus IX83	100×/1.3 UPLFLN	Andor Zyla 4.2+	180 mm SWTLU-C
3	Leica DMi8	100×/1.47 HCX PLAPO TIRF	Andor Zyla 4.2+	200 mm 11525408

Data processing methods

We processed all of the data presented here using SIMToolbox, an open source, user friendly, and freely available program which our group developed for processing SIM data [36]. SIMToolbox, sample data, and complete documentation are freely available (http://mmtg.fel.cvut.cz/SIMToolbox). SIMToolbox is capable of OS-SIM [10,37], SR-SIM [8,9], and MAP-SIM [30] methods. See the supplementary information for additional details about these methods.

Resolution measurements - spatial domain method

Previously, we used microscopy setup 1 (Olympus IX71) to measure spatial resolution by averaging spatial measurements from fifty individual 100 nm fluorescent beads [30]. We used a 100×/1.40 NA oil immersion objective and 460 nm LED excitation (emission 500 - 550 nm). A 19 × 19 pixel region of interest (ROI) was selected around each bead in both the widefield and MAP-SIM images. The ROIs were then registered with sub-pixel accuracy using normalized cross-correlation. Each ROI was fit with a Gaussian function and the full width at half maximum (FWHM) was determined in the axial and lateral directions. Figure 2 shows the resulting averaged FWHM values and PSF cross-sections [30].

Insert Figure 2

Resolution measurements - frequency domain method

It is desirable to measure the actual resolution achieved in SIM images (or image sequences) of cells or tissues, but suitable structures are not always present in the images. We therefore developed a robust frequency domain method which can be used to measure resolution in any fluorescence microscopy image [50].

The power spectral density (PSD) describes the distribution of the power of a signal with respect to its frequency. The PSD of an image is the squared magnitude of its Fourier transform, and can be written as

$$\mathrm{PSD}(k,l) = \left|\mathcal{F}\left\{I(m,n)\right\}\right|^2 \quad (1)$$

where $\mathcal{F}$ represents the Fourier transform, *I(m,n)* is the image intensity, *m,n* indexes the rows and columns of the 2D image, respectively, and (*k,l*) are coordinates in the frequency domain. In polar coordinates, the circularly averaged PSD (PSD_{ca}) in frequency space with frequency q and angle θ is given as

$$\mathrm{PSD}_{ca} = 10 \cdot \log_{10}\left(\frac{1}{N_q}\sum_{\theta}\mathrm{PSD}(q,\theta)\right) \quad (2)$$

which averages PSD at spatial frequency q. N_q is the number of pixels at a particular frequency q. The resolution limit in real space corresponds to the cut-off frequency in Fourier space. Assuming a noiseless case, the cut-off frequency will be equal to the spatial frequency at which PSD_{ca} drops to zero. In practice, PSD_{ca} contains non-zero values over the whole frequency range caused by noise. The signal to noise ratio (SNR) in Fourier space is generally very low close to the cut-off frequency, which makes precise detection of the cut-off frequency challenging. For this we use a spectral subtraction method [50]. Assuming additive noise, in the frequency domain we can write

$$\tilde{X}(k) = Y(k) - E\left[\left|N(k)\right|\right] \quad (3)$$

where $Y, \tilde{X}$, and $E\left[\left|N(k)\right|\right]$ represent the noisy signal, the desired signal, and the noise spectrum estimate (expected noise spectrum), respectively. The noise spectrum $\left|N(k)\right|$ is estimated from the parts of signal where only noise is present. If the spatial sampling is high enough to fulfill the Nyquist–Shannon criterion and oversamples the resolution limit of the reconstructed SIM image, spatial frequencies close to the half of the sampling frequency do not contain useful signal and can be used for noise estimation. We varied the frequency cut-off threshold over the range $\left\langle 0.95 f_{\max}; f_{\max} \right\rangle$, estimated the level of noise for every threshold value, and obtained the mean and variance of the cut-off frequency (i.e. the resolution estimate). The $f_{\max}$ is given by $f_{\max} = f_s/2 = 1/2p_{xy}$, where f_s and p_{xy} are the sampling frequency and the backprojected pixel size, respectively.

Figure 3 shows the PSD_{ca} and corresponding resolution limit measured for the data shown in Fig. 5. Using our resolution estimation algorithm, we calculated a lateral spatial resolution of 294 nm for WF, and 141 nm for MAP-SIM. The measured resolution is in approximate agreement with our results measured on 100 nm fluorescent beads (Fig. 2).

Insert Figure 3

Imaging live cells, fixed cells, and tissues with SIM

To demonstrate the utility of our approach in imaging live cells, we imaged U2-OS cells that had been transfected with GFP-tagged lysosomal associated membrane protein (LAMP1-GFP). LAMP1 is a highly glycosylated protein which is found on the surface of lysosomes and in the plasma membrane [51]. Fig. 4 shows widefield, OS-SIM, and MAP-SIM images of U2-OS cells expressing LAMP1-GFP, and the fast Fourier transform (FFT) of each image. The dotted circles in Fig. 4(d-f) show the approximate limit of resolution in each image. We found that, in addition to lysosomal expression, LAMP1-GFP is also present in high concentrations in the plasma membrane of U2-OS cells.

In this experiment, we acquired SIM image sequences with an exposure time of 25 ms, a raw imaging rate of 40 Hz. We used a SIM pattern with 11 phases (pattern period in the sample plane 1.5 μm) and a single angle (0º with respect to the camera), acquiring 3982 total frames, resulting in 472 processed frames (see table 1). The imaging rate of processed result frames was therefore 3.6 Hz. The full image sequence is available at http://mmtg.fel.cvut.cz/mapsimlive_suppl/ and also available at GigaDB[52]**.** We further analyzed this data as shown in the supplementary material (Figure S2-S3).

Insert Figure 4

We next imaged live A431 cells which we labeled with the fluorescent lipid DiI-C16. In this experiment we acquired SIM image sequences with an exposure time of 100 ms, a raw imaging rate of 10 Hz. We used a SIM pattern with 24 total phases and four angles (see table 1). This data is shown in Figure 5.

Insert Figure 5

Figure 6 shows SIM imaging of fixed tissues, in this case the seminiferous tubule of the rabbit stained with hematoxylin and eosin.

Insert Figure 6

Figure 7 shows SIM imaging of fixed HEPG2 cells expressing H4-Dendra, a nuclear marker. We also stained the cells with Atto 532-phalloidin to label the actin cytoskeleton.

Insert Figure 7

Figure 8 shows SIM imaging of fixed BPAE cells labeled with Alexa 488-phalloidin and mitotracker CMXRos to visualize the actin cytoskeleton and mitochondria, respectively.

Insert Figure 8

5. Discussion

SIM results sometimes suffer from artifacts related to the illumination pattern. The artifacts, which can be severe and are a cause for concern, can be due to several factors including illumination pattern phase instability and pattern distortion because of refractive index mismatch between the sample and the immersion fluid. In our hands, MAP-SIM results do not suffer from detectable patterned artifacts, Fig. 4(c), and the FFT of the MAP-SIM result is free of noticeable spurious peaks, Fig. 4(f). We attribute this to several factors, primarily the use of incoherent illumination together with a FLCOS microdisplay for pattern generation. This, combined with precise synchronization of the SIM system helps eliminate patterned artifacts. Additional artifacts in SIM images can arise due to the detector. In sCMOS cameras like the one we used, each pixel reads out through its own amplifier and as such, each pixel exhibits a different gain. While very minor, such artifacts can be corrected using a variance stabilization method as has been introduced for single molecule localization microscopy [53].

There are several other advantages to the use incoherent illumination in SIM, including removing the need for a pupil plane mask to block unwanted diffraction orders. Also, incoherent imaging of a microdisplay for pattern formation means that the pattern spatial frequency in the sample plane does not depend on the wavelength of the light which is used. On the other hand, in incoherent illumination SIM such as we used here, the contrast of the illumination pattern decreases with increasing spatial frequency according to the incoherent optical transfer function [54]. In coherent illumination SIM [8,9,11], the coherent optical transfer function applies [54], and so the pattern contrast does not decrease with increasing spatial frequency. This means that coherent illumination SIM can more efficiently mix high resolution information from outside the frequency limit into the detection passband of the microscope, thereby potentially achieving better resolution than what we achieved in this work. We achieved a lateral

resolution enhancement factor of ~1.8 (Fig. 2), whereas a factor of 2.0 is expected for coherent illumination SIM.

The FLCOS microdisplay (and vendor-supplied microdisplay-timing program) we used can display an illumination pattern and switch to the next pattern in the sequence in 1.14 ms, allowing unprocessed SIM images to be acquired at rates of approximately 875 Hz. However, such rapid imaging is not useful if the reconstructed SIM images are of poor quality, for example if they suffer from low signal to noise ratios. Specifying the fastest possible acquisition rate is inadequate without consideration of the resolution and SNR of the results. Our resolution analysis shown in Figs. 2-4 (see also supplementary figure 4) uses measured quantities to evaluate SIM results and thereby helps to make realistic conclusions about imaging speeds.

6. Re-use potential

The presented SIM datasets can be reused in several ways. Researchers investigating SIM reconstruction algorithms can use the datasets to compare their results with those presented here, including the newer method MAP-SIM. Also, the data may be further analyzed in other ways. One possibility is shown in the supplementary material (part 2: single particle tracking experiments in LAMP1-GFP cells.) Here, we used single particle tracking methods to study the mobility of lysosomes within U2-OS cells.

Availability of source code and requirements

Project name: SIMToolbox v1.3

Project home page: http://mmtg.fel.cvut.cz/SIMToolbox/

Operating system: platform independent

Programming language: MATLAB

License: GNU General Public License v3.0

Detailed software compatibility notes

The SIMToolbox GUI was compiled with MATLAB 2015a and tested in Windows 7 and 8. The GUI is a stand-alone program and does not require MATLAB to be installed. To use the MATLAB functions

within SIMToolbox (i.e., without the GUI), MATLAB must be installed. The functions were mainly developed with 64 bit MATLAB versions 2012b, 2014a, 2015a in Windows 7. When using SIMToolbox functions without the GUI, the MATLAB "Image Processing Toolbox" is required. SIMToolbox also requires the "MATLAB YAML" package to convert MATLAB objects to/from YAML file format. Note that this package is installed automatically when using the GUI.

Availability of data

All raw and analyzed data is available in the *GigaScience* GigaDB database [52].

Abbreviations

GFP: green fluorescent protein; GUI: graphical user interface; NA: numerical aperture; PSF: point spread function; PSD: power spectral density; PSDca, circularly averaged power spectral density; SBR: signal to background ratio; SIM: structured illumination microscopy; SNR: signal to noise ratio; WF: wide field.

Ethics approval and consent to participate

Not applicable

Consent for publication

Not applicable

Competing interests

The authors declare that they have no competing interests.

Funding

This work was supported by the National Institutes of Health grant number 1R15GM128166-01. This work was also supported by the UCCS center for the University of Colorado BioFrontiers Institute, by the Czech Science Foundation, and by Czech Technical University in Prague (grant number SGS18/141/OHK3/2T/13). T.L. acknowledges a SCIEX scholarship (project code 13.183). The funding sources had no involvement in study design; in the collection, analysis and interpretation of data; in the writing of the report; or in the decision to submit the article for publication. This material is based in part upon work supported by the National Science Foundation under Grant Number 1727033. Any opinions,

findings, and conclusions or recommendations expressed in this material are those of the authors and do not necessarily reflect the views of the National Science Foundation.

Author Contributions

TL: analyzed data, developed computer code

JP: analyzed data, developed computer code

KF: supervised research

KS: analyzed data

JB: acquired data

GH: conceived project, acquired data, analyzed data, supervised research, wrote the paper

Acknowledgements

The authors thank Dr. Donna Arndt-Jovin and Dr. Tomas Jovin of the Max Planck Institute for Biophysical Chemistry (Göttingen, Germany) for the A431 cells. The authors thank Viola Hausnerová and Christian Lanctôt of Charles University in Prague (Prague, Czech Republic), for the LAMP1-GFP cells. The authors thank Pavel Křížek, Zdeněk Švindrych, and Martin Ovesný for help with data acquisition, microscopy development, programming, and data analysis.

References

1. E. Betzig, G. H. Patterson, R. Sougrat, O. W. Lindwasser, S. Olenych, J. S. Bonifacino, M. W. Davidson, J. Lippincott-Schwartz, and H. F. Hess, "Imaging intracellular fluorescent proteins at nanometer resolution," Science **313**, 1642–5 (2006).

2. S. T. Hess, T. P. K. Girirajan, and M. D. Mason, "Ultra-high resolution imaging by fluorescence photoactivation localization microscopy," Biophys. J. **91**, 4258–4272 (2006).

3. M. J. Rust, M. Bates, and X. Zhuang, "Sub-diffraction-limit imaging by stochastic optical reconstruction microscopy (STORM)," Nat. Methods **3**, 793–795 (2006).

4. M. Heilemann, S. van de Linde, M. Schüttpelz, R. Kasper, B. Seefeldt, A. Mukherjee, P. Tinnefeld, and M. Sauer, "Subdiffraction-resolution fluorescence imaging with conventional

fluorescent probes," Angew. Chemie Int. Ed. **47**, 6172–6176 (2008).

5. T. Dertinger, R. Colyer, G. Iyer, S. Weiss, and J. Enderlein, "Fast, background-free, 3D super-resolution optical fluctuation imaging (SOFI)," Proc. Natl. Acad. Sci. U. S. A. **106**, 22287–22292 (2009).

6. S. Geissbuehler, N. L. Bocchio, C. Dellagiacoma, C. Berclaz, M. Leutenegger, and T. Lasser, "Mapping molecular statistics with balanced super-resolution optical fluctuation imaging (bSOFI)," Opt. Nanoscopy **1**, 4 (2012).

7. S. W. Hell and J. Wichmann, "Breaking the diffraction resolution limit by stimulated emission: stimulated-emission-depletion fluorescence microscopy," Opt. Lett. **19**, 780 (1994).

8. R. Heintzmann and C. Cremer, "Laterally modulated excitation microscopy: improvement of resolution by using a diffraction grating," Proc. SPIE **3568**, 185–196 (1998).

9. M. G. L. Gustafsson, "Surpassing the lateral resolution limit by a factor of two using structured illumination microscopy," J. Microsc. **198**, 82–87 (2000).

10. M. A. A. Neil, R. Juškaitis, and T. Wilson, "Method of obtaining optical sectioning by using structured light in a conventional microscope," Opt. Lett. **22**, 1905–1907 (1997).

11. M. G. L. Gustafsson, L. Shao, P. M. Carlton, C. J. R. Wang, I. N. Golubovskaya, W. Z. Cande, D. A. Agard, and J. W. Sedat, "Three-dimensional resolution doubling in widefield fluorescence microscopy by structured illumination," Biophys. J. **94**, 4957–4970 (2008).

12. P. Kner, B. B. Chhun, E. R. Griffis, L. Winoto, and M. G. L. Gustafsson, "Super-resolution video microscopy of live cells by structured illumination," Nat. Methods **6**, 339–342 (2009).

13. L. M. Hirvonen, K. Wicker, O. Mandula, and R. Heintzmann, "Structured illumination microscopy of a living cell," Eur. Biophys. J. **38**, 807–812 (2009).

14. L. Shao, P. Kner, E. H. Rego, and M. G. L. Gustafsson, "Super-resolution 3D microscopy of live whole cells using structured illumination," Nat. Methods **8**, 1044–1046 (2011).

15. L. Schermelleh, P. M. Carlton, S. Haase, L. Shao, L. Winoto, P. Kner, B. Burke, M. C. Cardoso,

D. A. Agard, M. G. L. Gustafsson, H. Leonhardt, and J. W. Sedat, "Subdiffraction multicolor imaging of the nuclear periphery with 3D structured illumination microscopy," Science **320**, 1332–1336 (2008).

16. R. Fiolka, L. Shao, E. H. Rego, M. W. Davidson, and M. G. L. Gustafsson, "Time-lapse two-color 3D imaging of live cells with doubled resolution using structured illumination," Proc. Natl. Acad. Sci. U. S. A. **109**, 5311–5315 (2012).

17. A. G. York, S. H. Parekh, D. D. Nogare, R. S. Fischer, K. Temprine, M. Mione, A. B. Chitnis, C. A. Combs, and H. Shroff, "Resolution doubling in live, multicellular organisms via multifocal structured illumination microscopy," Nat. Methods **9**, 749–754 (2012).

18. M. Ingaramo, A. G. York, P. Wawrzusin, O. Milberg, A. Hong, R. Weigert, H. Shroff, and G. H. Patterson, "Two-photon excitation improves multifocal structured illumination microscopy in thick scattering tissue," Proc. Natl. Acad. Sci. U. S. A. **111**, 5254–9 (2014).

19. A. G. York, P. Chandris, D. D. Nogare, J. Head, P. Wawrzusin, R. S. Fischer, A. Chitnis, and H. Shroff, "Instant super-resolution imaging in live cells and embryos via analog image processing.," Nat. Methods **10**, 1122–6 (2013).

20. M. Schropp and R. Uhl, "Two-dimensional structured illumination microscopy," J. Microsc. **256**, 23–36 (2014).

21. M. Schropp, C. Seebacher, and R. Uhl, "XL-SIM: Extending Superresolution into Deeper Layers," Photonics **4**, 33 (2017).

22. T. A. Planchon, L. Gao, D. E. Milkie, M. W. Davidson, J. A. Galbraith, G. Catherine, C. G. Galbraith, and E. Betzig, "Rapid three-dimensional isotropic imaging of living cells using Bessel beam plane illumination," Nat. Methods **8**, 417–423 (2011).

23. P. J. Keller, A. D. Schmidt, A. Santella, K. Khairy, Z. Bao, J. Wittbrodt, and E. H. K. Stelzer, "Fast, high-contrast imaging of animal development with scanned light sheet-based structured-illumination microscopy," Nat Meth **7**, 637–642 (2010).

24. L. Gao, L. Shao, C. C. D. Higgins, J. S. J. Poulton, M. Peifer, M. W. Davidson, X. Wu, B. Goldstein, and E. Betzig, "Noninvasive imaging beyond the diffraction limit of 3D dynamics in thickly fluorescent specimens," Cell **151**, 1370–1385 (2012).

25. B.-C. Chen, W. R. Legant, K. Wang, L. Shao, D. E. Milkie, M. W. Davidson, C. Janetopoulos, X. S. Wu, J. A. Hammer, Z. Liu, B. P. English, Y. Mimori-Kiyosue, D. P. Romero, A. T. Ritter, J. Lippincott-Schwartz, L. Fritz-Laylin, R. D. Mullins, D. M. Mitchell, J. N. Bembenek, A.-C. Reymann, R. Bohme, S. W. Grill, J. T. Wang, G. Seydoux, U. S. Tulu, D. P. Kiehart, and E. Betzig, "Lattice light-sheet microscopy: Imaging molecules to embryos at high spatiotemporal resolution," Science **346**, 1257998 (2014).

26. D. Li, L. Shao, B.-C. Chen, X. Zhang, M. Zhang, B. Moses, D. E. Milkie, J. R. Beach, J. A. Hammer, M. Pasham, T. Kirchhausen, M. A. Baird, M. W. Davidson, P. Xu, and E. Betzig, "Extended-resolution structured illumination imaging of endocytic and cytoskeletal dynamics," Science **349**, aab3500 (2015).

27. S. Dong, P. Nanda, R. Shiradkar, K. Guo, and G. Zheng, "High-resolution fluorescence imaging via pattern-illuminated Fourier ptychography.," Opt. Express **22**, 20856–70 (2014).

28. F. Orieux, E. Sepulveda, V. Loriette, B. Dubertret, and J.-C. Olivo-Marin, "Bayesian estimation for optimized structured illumination microscopy.," IEEE Trans. Image Process. **21**, 601–14 (2012).

29. T. Lukeš, G. M. G. M. Hagen, P. Křížek, Z. Švindrych, K. Fliegel, M. Klíma, P. Krížek, Z. Švindrych, K. Fliegel, and M. Klíma, "Comparison of image reconstruction methods for structured illumination microscopy," Proc. SPIE **9129**, 91293J (2014).

30. T. Lukeš, P. Křížek, Z. Švindrych, J. Benda, M. Ovesný, K. Fliegel, M. Klíma, and G. M. Hagen, "Three-dimensional super-resolution structured illumination microscopy with maximum a posteriori probability image estimation," Opt. Express **22**, 29805–17 (2014).

31. E. Mudry, K. Belkebir, J. Girard, J. Savatier, E. Le Moal, C. Nicoletti, M. Allain, and A.

Sentenac, "Structured illumination microscopy using unknown speckle patterns," Nat. Photonics **6**, 312–315 (2012).

32. X. Huang, J. Fan, L. Li, H. Liu, R. Wu, Y. Wu, L. Wei, H. Mao, A. Lal, P. Xi, L. Tang, Y. Zhang, Y. Liu, S. Tan, and L. Chen, "Fast, long-term, super-resolution imaging with Hessian structured illumination microscopy," Nat. Biotechnol. (2018).

33. V. Perez, B. J. Chang, and E. H. K. Stelzer, "Optimal 2D-SIM reconstruction by two filtering steps with Richardson-Lucy deconvolution," Sci. Rep. **6**, 37149 (2016).

34. K. Chu, P. J. McMillan, Z. J. Smith, J. Yin, J. Atkins, P. Goodwin, S. Wachsmann-Hogiu, and S. Lane, "Image reconstruction for structured-illumination microscopy with low signal level," Opt. Express **22**, 8687 (2014).

35. N. Chakrova, B. Rieger, and S. Stallinga, "Deconvolution methods for structured illumination microscopy," J. Opt. Soc. Am. A **33**, B12 (2016).

36. P. Křížek, T. Lukeš, M. Ovesný, K. Fliegel, and G. M. Hagen, "SIMToolbox: A MATLAB toolbox for structured illumination fluorescence microscopy," Bioinformatics **32**, 318–320 (2015).

37. P. Křížek, I. Raška, and G. M. Hagen, "Flexible structured illumination microscope with a programmable illumination array," Opt. Express **20**, 24585 (2012).

38. G. M. Hagen, W. Caarls, M. Thomas, A. Hill, K. A. Lidke, B. Rieger, C. Fritsch, B. van Geest, T. M. Jovin, and D. J. Arndt-Jovin, "Biological applications of an LCoS-based programmable array microscope," Proc. SPIE **64410S**, 1–12 (2007).

39. C. A. Werley, M.-P. Chien, and A. E. Cohen, "An ultrawidefield microscope for high-speed fluorescence imaging and targeted optogenetic stimulation," Biomed. Opt. Express **8**, 5794 (2017).

40. D. Dan, M. Lei, B. Yao, W. Wang, M. Winterhalder, A. Zumbusch, Y. Qi, L. Xia, S. Yan, Y. Yang, P. Gao, T. Ye, and W. Zhao, "DMD-based LED-illumination super-resolution and optical sectioning microscopy.," Sci. Rep. **3**, 1116 (2013).

41. P. Křížek and G. M. Hagen, "Spatial light modulators in fluorescence microscopy," in *Microscopy: Science, Technology, Applications and Education*, A. Méndez-Vilas, ed., 4th ed. (Formatex, 2010), Vol. 2, pp. 1366–1377.

42. G. M. Hagen, W. Caarls, K. A. Lidke, A. H. B. De Vries, C. Fritsch, B. G. Barisas, D. J. Arndt-Jovin, and T. M. Jovin, "Fluorescence recovery after photobleaching and photoconversion in multiple arbitrary regions of interest using a programmable array microscope," Microsc. Res. Tech. **72**, 431–440 (2009).

43. L. Song, H.-W. Lu-Walther, R. Förster, A. Jost, M. Kielhorn, J. Zhou, and R. Heintzmann, "Fast structured illumination microscopy using rolling shutter cameras," Meas. Sci. Technol. **27**, 055401 (2016).

44. Z. Cvačková, M. Mašata, D. Staněk, H. Fidlerová, and I. Raška, "Chromatin position in human HepG2 cells: although being non-random, significantly changed in daughter cells.," J. Struct. Biol. **165**, 107–17 (2009).

45. L. J. Young, F. Ströhl, and C. F. Kaminski, "A Guide to Structured Illumination TIRF Microscopy at High Speed with Multiple Colors," J. Vis. Exp. e53988–e53988 (2016).

46. R. Förster, H.-W. Lu-Walther, A. Jost, M. Kielhorn, K. Wicker, and R. Heintzmann, "Simple structured illumination microscope setup with high acquisition speed by using a spatial light modulator," Opt. Express **22**, 20663–77 (2014).

47. H.-W. Lu-Walther, M. Kielhorn, R. Förster, A. Jost, K. Wicker, and R. Heintzmann, "fastSIM: a practical implementation of fast structured illumination microscopy," Methods Appl. Fluoresc. **3**, 014001 (2015).

48. T. C. Schlichenmeyer, M. Wang, K. N. Elfer, and J. Q. Brown, "Video-rate structured illumination microscopy for high-throughput imaging of large tissue areas.," Biomed. Opt. Express **5**, 366–77 (2014).

49. S. R. Kantelhardt, W. Caarls, A. H. B. de Vries, G. M. Hagen, T. M. Jovin, W. Schulz-Schaeffer,

V. Rohde, A. Giese, and D. J. Arndt-Jovin, "Specific visualization of glioma cells in living low-grade tumor tissue," PLoS One **5**, 1–11 (2010).

50. J. Pospíšil, K. Fliegel, and M. Klíma, "Assessing resolution in live cell structured illumination microscopy," in *Proceedings of SPIE - The International Society for Optical Engineering*, P. Páta and K. Fliegel, eds. (SPIE, 2017), Vol. 10603, p. 39.

51. A. K. Agarwal, N. Srinivasan, R. Godbole, S. K. More, S. Budnar, R. P. Gude, and R. D. Kalraiya, "Role of tumor cell surface lysosome-associated membrane protein-1 (LAMP1) and its associated carbohydrates in lung metastasis.," J. Cancer Res. Clin. Oncol. **141**, 1563–74 (2015).

52. Pospíšil J; Lukeš T; Bendesky J; Fliegel K; Spendier K; Hagen GM (2018): Supporting data for "Imaging tissues and cells beyond the diffraction limit with structured illumination microscopy and Bayesian image reconstruction" GigaScience Database. http://dx.doi.org/10.5524/100514

53. F. Huang, T. M. P. Hartwich, F. E. Rivera-Molina, Y. Lin, W. C. Duim, J. J. Long, P. D. Uchil, J. R. Myers, M. A. Baird, W. Mothes, M. W. Davidson, D. Toomre, and J. Bewersdorf, "Video-rate nanoscopy using sCMOS camera-specific single-molecule localization algorithms.," Nat. Methods **10**, 1–9 (2013).

54. J. W. Goodman, "Frequency Analysis of Optical Imaging Systems," in *Introduction to Fourier Optics*, 2nd ed. (McGraw-HIll Int., 1968), pp. 126–171.

55. M. Geissbuehler and T. Lasser, "How to display data by color schemes compatible with red-green color perception deficiencies.," Opt. Express **21**, 9862–74 (2013).

FIGURE CAPTIONS

Figure 1: Structured illumination microscope setup, which we used with different microscope bodies and cameras. See text and table 2 for details.

Figure 2: Measurements of the spatial resolution on a sample of fluorescent beads. Cross-sections of the PSF are obtained by averaging measurements over 50 beads along lateral and axial directions.

Figure 3: Resolution analysis and normalized power spectral density (PSD) measured on a selected image from the data in Fig. 5. The results indicate a circularly-averaged PSD lateral spatial resolution of 294 nm for WF, and 141 nm for MAP-SIM, in approximate agreement with the analysis in Fig. 4(d-f).

Figure 4: Imaging live cells beyond the difraction limit with MAP-SIM. U2-OS cells expressing LAMP1-GFP were imaged using the LCOS-based SIM system. Subsequent processing using OS-SIM or MAP-SIM methods. (a) WF, (b) OS-SIM, (c) MAP-SIM, (d) FFT of WF, (e) FFT of OS-SIM, (f) FFT of MAP-SIM. The images were individually scaled for presentation. The dotted cirular lines indicate the approximate resolution achieved in each image according to analysis of the FFT. The full image sequence is available at http://mmtg.fel.cvut.cz/mapsimlive_suppl/.

Figure 5: Imaging live cells beyond the difraction limit with SIM. A431 cells labeled with DiI-C16 were imaged using the LCOS-based SIM system. Subsequent processing using SR-SIM or MAP-SIM methods. (a) WF, (c) SR-SIM, (e) MAP-SIM. (b), (d), and (f) each show a zoom-in of the region indicated in (a). (g) shows the SIM illumination pattern in one of the four angles used. (h) shows a FFT of the image in (g). The images were individually scaled for visualization purposes. Each is a maximum intenstiy projection of 3 Z positions (spacing 400 nm (except for g and h which show a single Z-position).

Figure 6: Imaging animal tissues using the LCOS-based SIM system and subsequent processing using OS-SIM or MAP-SIM methods. Seminiferous tubule of the rabbit stained with hematoxylin and eosin. (a) WF, (c) OS-SIM, (e) MAP-SIM. (b), (d), and (f) each show a zoom-in of the region indicated in (a). (g) shows the SIM illumination pattern in one of the four angles used. (h) MAP-SIM depth-coded using the lookup table isolum [55]. The images were individually scaled for visualization purposes. Each is a maximum intenstiy projection of 31 Z-positions (spacing 300 nm (except for (a, b, g) which show 1 Z-position).

Figure 7: SIM imaging of fixed HEP-G2 cells expressing Dendra2-H4 (nucleus) and labeled with Atto-532 phalloidin. (a) WF, (c) SR-SIM, (e) MAP-SIM. (b), (d), and (f) each show a zoom-in of the region

indicated in (a). (g) shows the SIM illumination pattern in one of the four angles used. (h) shows a FFT of the image in (g). The images were individually scaled for visualization purposes. Each is a maximum intenstiy projection of 22 Z-positions (spacing 200 nm (except for a, b, g and h which show 1 Z-position).

Figure 8: 2D SIM imaging of fixed BPAE cells labeled with Alexa 488-phalloidin (actin) and mitotracker CMXRos (mitochondria). (a) WF, (b) MAPSIM.

FIG 1

Click here to access/download;Figure;FIG 1.docx

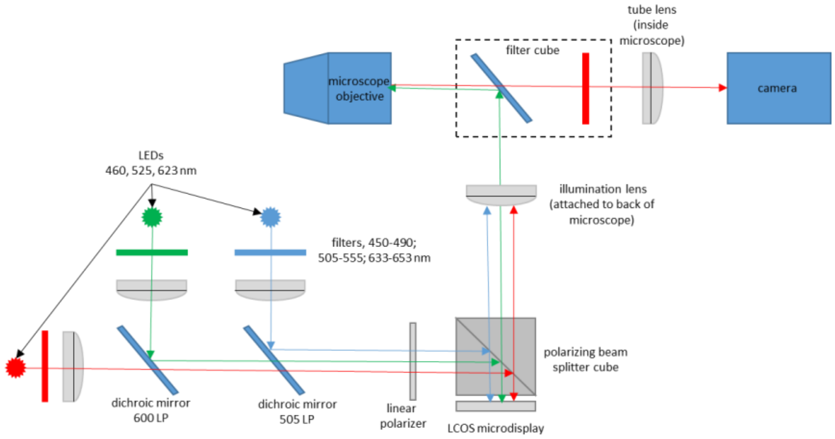

FIG 2

Click here to access/download;Figure;FIG 2.docx

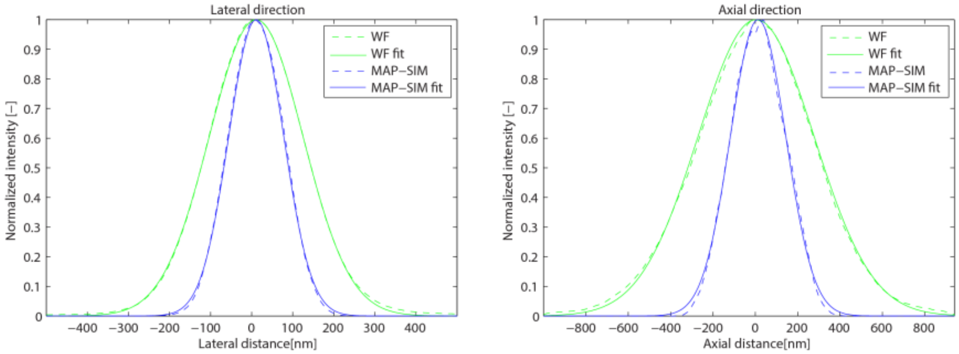

Lateral resolution
$FWHM_{WF}$ = (258 ± 12) nm
$FWHM_{MAP\text{-}SIM}$ = (144 ± 7) nm

Axial resolution
$FWHM_{WF}$ = (632 ± 44) nm
$FWHM_{MAP\text{-}SIM}$ = (299 ± 50) nm

FIG 3

Click here to access/download;Figure;FIG 3.docx

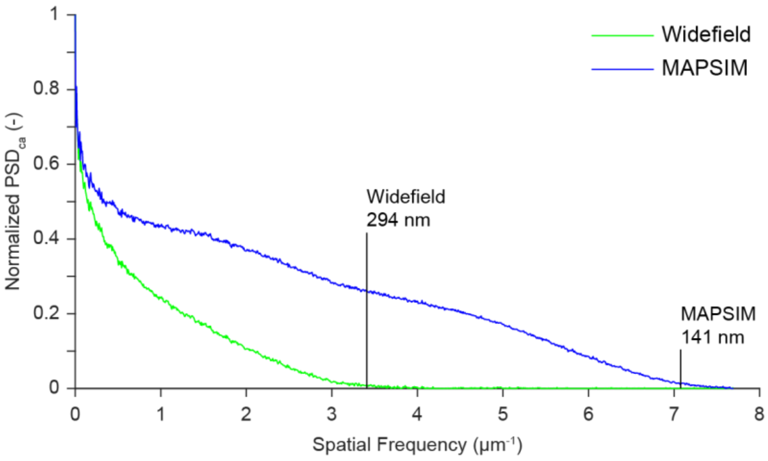

Click here to access/download;Figure;FIG 4.docx

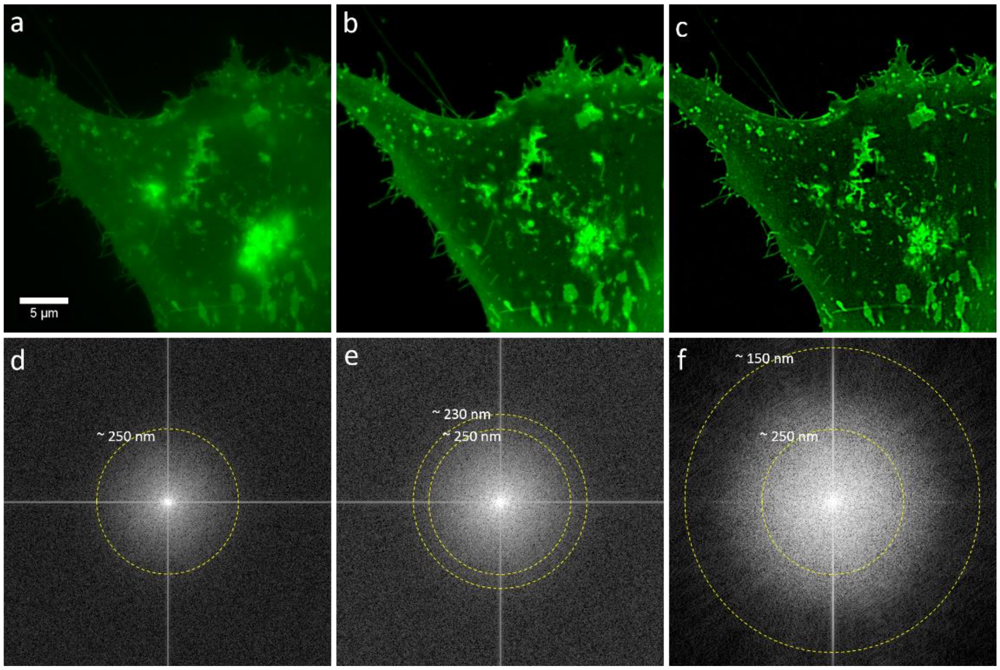

Click here to access/download;Figure;FIG 5 revised.docx

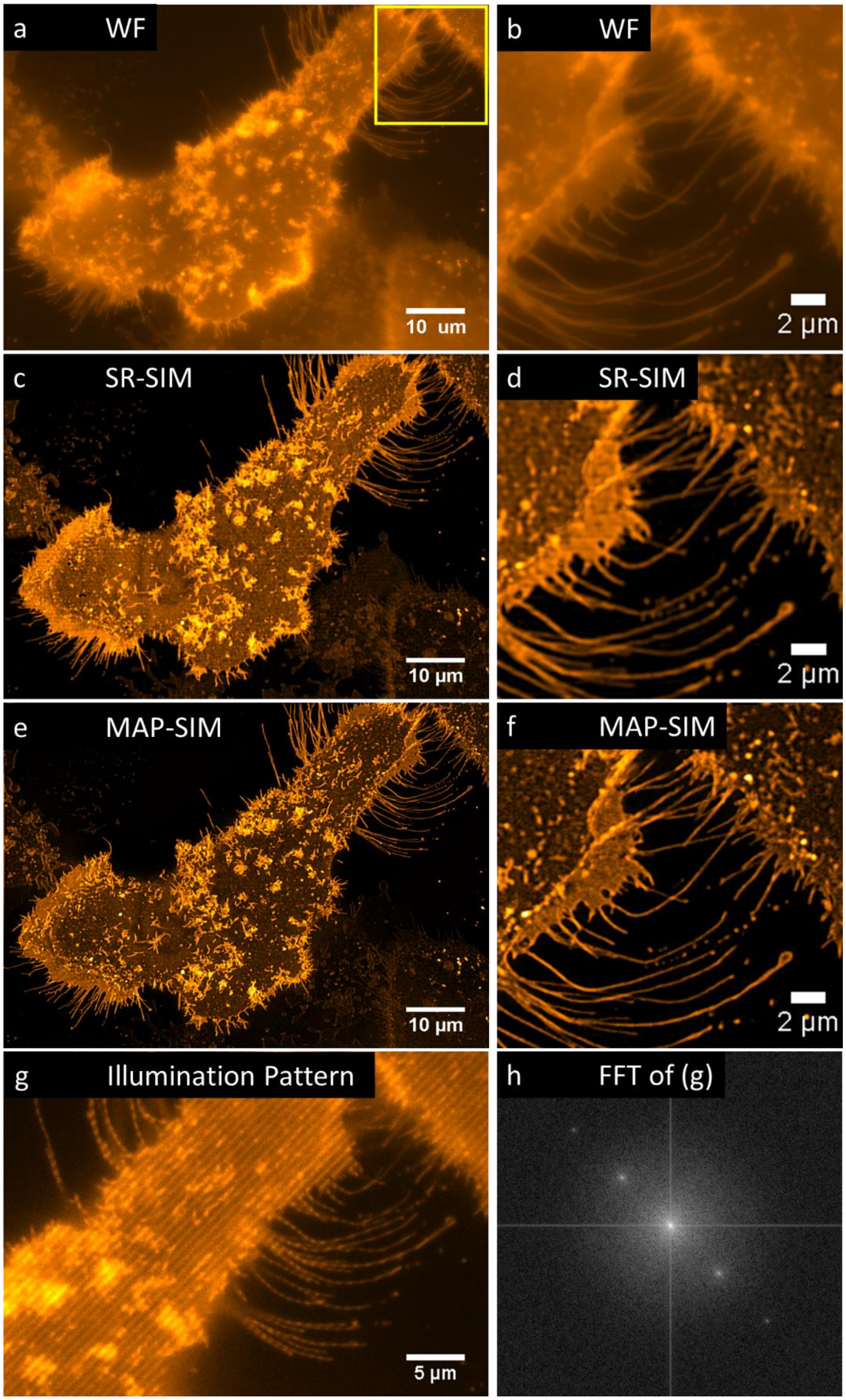

FIG 6 Click here to access/download;Figure;FIG 6.docx

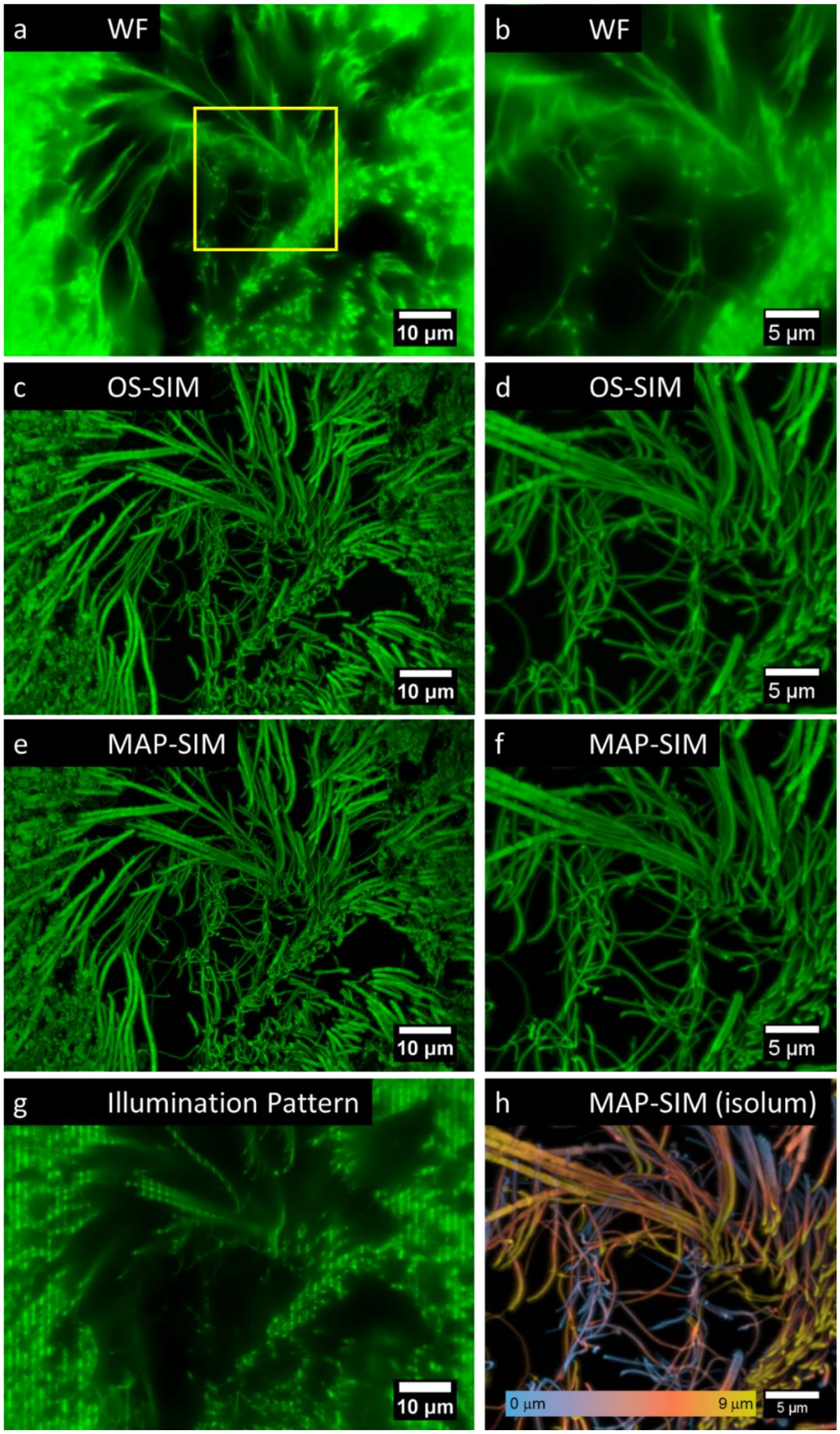

FIG 7

Click here to access/download;Figure;FIG 7 revised.docx

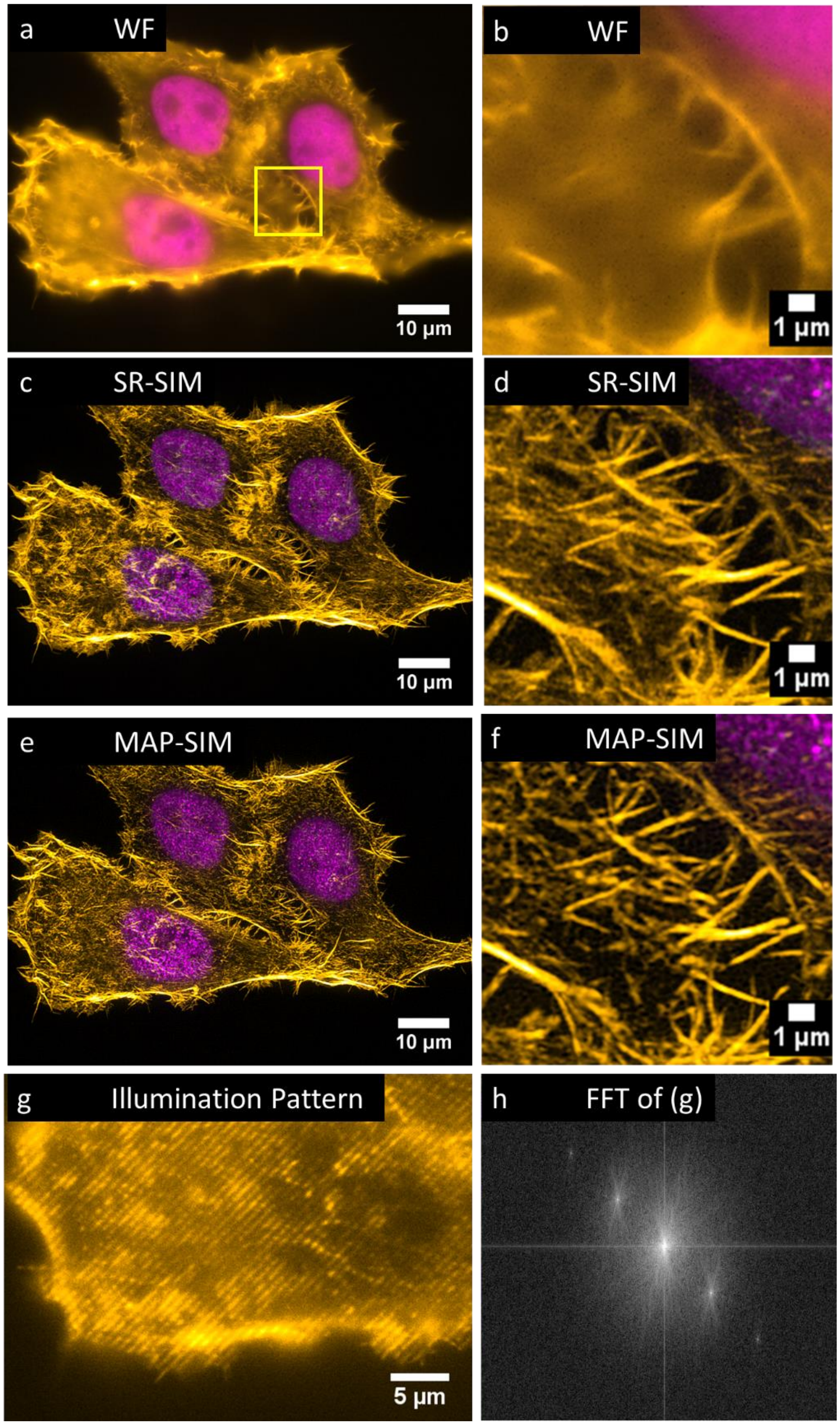

FIG 8

Click here to access/download;Figure;FIG 8.docx

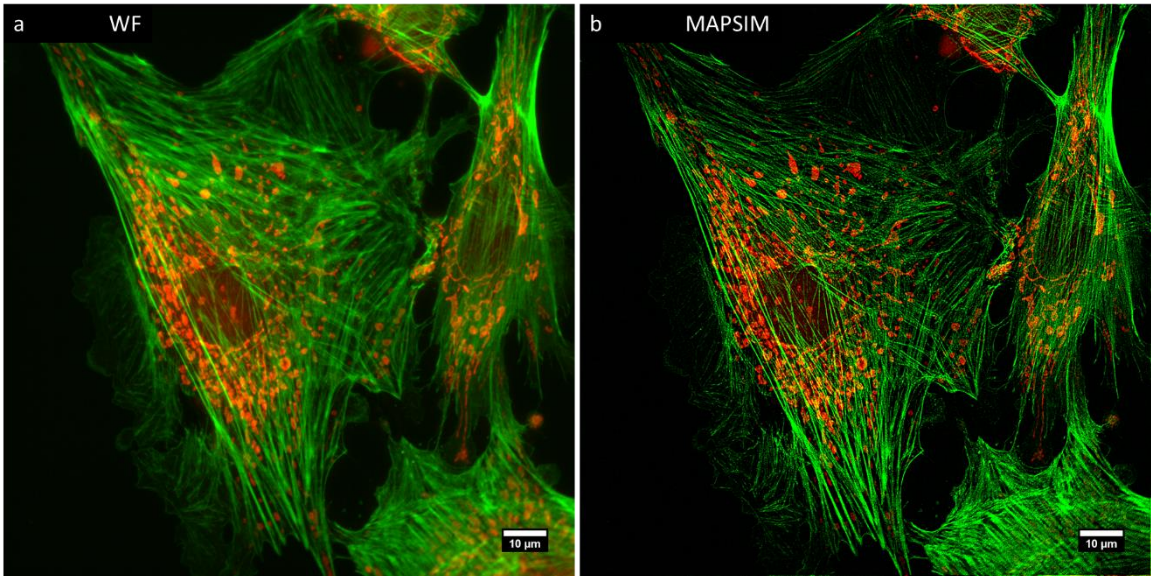

Click here to access/download
Supplementary Material
Hagen-SIM Supplementary-resubmit.docx

Personal Cover

Click here to access/download;Personal Cover;Hagen letter to editor - resubmit.docx

University of Colorado Colorado Springs

Dr. Guy M. Hagen

Biofrontiers

1420 Austin Bluffs Pkwy.

Colorado Springs, CO 80918

Tel. 719-255-3692

ghagen@uccs.edu

Dear Editor:

We would like to resubmit our manuscript entitled “Imaging tissues and cells beyond the diffraction limit with structured illumination microscopy and Bayesian image reconstruction” for consideration in *GigaScience* as a data note.

We would like to thank the referees for their very positive reviews of the paper. Reviewer 1 had a minor point of concern:

“One minor point the authors may want to address is the difference between coherent interference illumination and the incoherent pattern illumination employed in the manuscript. It seems that interference illumination can generate a sin pattern with highest contrast at cutoff frequency while incoherent pattern illumination may suffer from the gradually cutoff of the incoherent OTF.”

We thank the reviewer for this comment. The reviewer is correct about this, and we have written some comments and added a new reference in the discussion which should clear up this matter. The modified paragraph now reads:

“There are several other advantages to the use incoherent illumination in SIM, including removing the need for a pupil plane mask to block unwanted diffraction orders. Also, incoherent imaging of a microdisplay for pattern formation means that the pattern spatial frequency in the sample plane does not depend on the wavelength of the light which is used. On the other hand, in incoherent illumination SIM such as we used here, the contrast of the illumination pattern decreases with increasing spatial frequency according to the incoherent optical transfer function [53]. In coherent illumination SIM [8,9,11], the coherent optical transfer function applies [53], and so the pattern contrast does not decrease with increasing spatial frequency. This means that coherent illumination SIM can more efficiently mix high resolution information from outside the frequency limit into the detection passband of the microscope, thereby potentially achieving better resolution than what we achieved in this work. We achieved a lateral resolution enhancement factor of ~1.8 (Fig. 2), whereas a factor of 2.0 is expected for coherent illumination SIM.”

In addition to this, we have fixed a few typographical errors.

We hope that the paper will now be acceptable for publication.

Sincerely,

Guy Hagen

Guy M. Hagen
