# Supplementary material for: Imaging tissues and cells beyond the diffraction limit with structured illumination microscopy and Bayesian image reconstruction
Source: Gigascience. 2018 Aug 23;8(1):giy126. doi: 10.1093/gigascience/giy126 (PMC6325271; doi:10.1093/gigascience/giy126)
Supplement: Supplemental File [file giy126_supplemental_file.docx]

**Imaging tissues and cells beyond the diffraction limit with structured illumination microscopy and
 Bayesian image reconstruction**

**Jakub Pospíšil^1^, Tomáš Lukeš^1,2^, Justin Bendesky^3^, Karel Fliegel^1^,
Kathrin Spendier^3,4^, and Guy M. Hagen^3*^**

^1^Department of Radioelectronics, Faculty of Electrical Engineering, Czech Technical University in Prague, Technická 2, 16627 Prague 6, Czech Republic

^2^Laboratory of Nanoscale Biology, École Polytechnique Fédérale de Lausanne, CH-1015 Lausanne, Switzerland

^3^UCCS center for the Biofrontiers Institute, University of Colorado at Colorado Springs, 1420 Austin Bluffs Parkway, Colorado Springs, Colorado, 80918, USA

^4^Department of Physics and Energy Science, University of Colorado at Colorado Springs, 1420 Austin Bluffs Parkway, Colorado Springs, Colorado, 80918, USA

^*^ ghagen@uccs.edu

**Supplementary Information**

**1. OS-SIM and MAP-SIM**

1.1 Optical Sectioning SIM (OS-SIM)

Several data processing methods are possible for generating optically sectioned images from SIM data (OS-SIM) [1,2]. The most familiar implementation of this technique was introduced in 1997 by Neil et al. [3]. Their method works by projecting a line illumination pattern onto a sample, followed by acquisition of a set of three widefield images with the pattern shifted by relative spatial phases 0, 2π/3, and 4π/3. An optically sectioned image can be recovered computationally as

$I_{OS-SIM}=\left[(I_{1}-I_{2})^{2}+(I_{1}-I_{3})^{2}+(I_{2}-I_{3})^{2}\right]^{1/2},$ (S1)

where *I_OS-SIM_* is optically sectioned image, and *I_1_, I_2_,* and *I_3_* are the three images acquired with different pattern positions. This is sometimes called a ‘square law’ method. If the sum of the individual SIM patterns results in homogeneous illumination, as is the case in our setups, a widefield image can be recovered from SIM data as an average of all images:

$I_{WF}=\frac{1}{N}\sum_{n=1}^{N} I_{n} .$ (S2)

This is the approach we used throughout this study to generate WF images.

In this study we used a method known as homodyne detection [1–3].

$I_{OS-SIM}=\left|\sum_{n=1}^{N} I_{n}\exp\left(2\pi\mathrm{i}\frac{n}{N}\right)\right| .$ (S3)

We previously showed that this processing method offers results with better optical sectioning than the square law method of Eq. S2 [2].

1.2 SIM with maximum a posteriori probability estimation

MAP-SIM has been described previously [4]. In this case, image acquisition in structured illumination microscopy can be described as

$y_{k}=HM_{k}x+ n_{k},$ (S4)

where *M_k_* represents the *k*-th illumination pattern, ***y****_k_* denotes a low-resolution (LR) image acquired using the *k*-th illumination pattern, ***x*** is an unknown, high-resolution (HR) image, and ***n****_k_* is additive noise. *H* is a matrix describing the convolution between the HR image and the point spread function (PSF) of the system. The position of the illumination patterns in the camera images were determined using a calibrated camera according to our previous work [2]. We model the PSF as an Airy disk, which in Fourier space leads to an optical transfer function (OTF) of the form [5]

$OTF\left(f\right)= \frac{1}{\pi}\left[2\cos^{-1}\left(\frac{f}{f_{c}}\right)-\text{sin}\left(2\cos^{-1}\left(\frac{f}{f_{c}}\right)\right)\right],$ (S5)

where ***f*** is the spatial frequency. We estimate the cut-off frequency *f_c_* by calculating the radial average of the power spectral density (PSD) of a widefield image of 100 nm fluorescent beads.

Using a Bayesian approach [4,6–10], high-resolution image estimation can be expressed as a minimization of a cost function according to

$x_{\text{HR-MAP}}=\mathop{\text{arg}\min}\limits_{x} \left[\sum_{k=1}^{K} \left\|y_{k}-HM_{k}x\right\|^{2}+\text{λ}\Gamma\left(x\right)\right]$. (S6)

The cost function in Eq. S6 consists of two terms. The first term describes the mean square error between the estimated HR image and the observed LR images. The second term is a regularization term. To ensure positivity and promote a smoothness condition on the HR image, we rely on quadratic regularization composed of finite difference approximations of the first order derivative at each pixel location [11]

$\Gamma\left(x\right)= \sum_{i} \left[\left(\Delta_{h}x\right)_{i}^{2} +\left(\Delta_{v}x\right)_{i}^{2} \right]$ , (S7)

where $\Delta_{h}$ and $\Delta_{v}$ are the finite difference operators along the horizontal and vertical direction of an image, and denotes the *i*-th element of a vector. The contribution of $\Gamma\left(x\right)$ is controlled by the parameter λ, a small positive constant defining the strength of the regularization (typically λ = 0.01). We solve Eq. (S6) using gradient descent methods.

1.3 Spectral merging

MAP estimation of high resolution images obtained with structured illumination enables reconstruction of high resolution images (HR-MAP) with details unresolvable in a widefield microscope. However, MAP estimation as described above does not suppress out of focus light. On the other hand, the homodyne detection method

$I_{OS-SIM}=x_{\text{LR-HOM}}=\left|\sum_{k=1}^{K} y_{k}\exp\left(2\pi\text{i}\frac{k}{K}\right)\right|$ (S8)

used in optical sectioning SIM (OS-SIM) [2,3] provides images (LR-HOM) with optical sectioning but with only a slight improvement in lateral resolution. Noting that the unwanted out of focus light is dominant at low spatial frequencies, we merge the LR-HOM and HR-MAP images in the frequency domain to obtain the final HR image (MAP-SIM). Frequency domain Gaussian low pass filtering is applied to the LR-HOM image and a complementary high pass filter is applied to the HR-MAP image. We use a weighting scheme which can be described by

, (S9)

where denotes the Fourier transform operator and its inverse, respectively, ***f*** is the spatial frequency, *σ* is the standard deviation of the Gaussian filter, and *β* is a weighting coefficient. Usually we set *β* = 0.85. We typically use a circularly symmetric cosine bell apodizing function to shape the final MAP-SIM spectrum before the final inverse FFT. The spectral merging method is shown schematically in figure S2.

Fig. S1. Schematic of spectral merging (a) Spatial frequencies in Fourier space, where *f_c_* is the cut off frequency for a WF microscope. (b) Power spectral density (PSD) in relation to the spatial frequency. (c) Blending frequency spectra of HR-MAP estimation and LR homodyne detection using low and high pass filters.

**2. Example of data re-use: Single particle tracking experiments in LAMP1-GFP cells**

2.1 The optical sectioning effect of SIM allows tracking of low signal to background ratio particles in LAMP1-GFP cells

Single particle tracking (SPT) is a computer enhanced microscopy method used to track the motion of biological molecules or vesicles [12–14]. In SPT, a particle trajectory is obtained from position coordinates over a series of time steps. There are three basic steps in single particle tracking analysis [15]. The first is detection of the particles in the raw data. This may be regarded as segmentation or feature detection. The second step is localization of the particle, usually accomplished by fitting a small region of interest (7×7 pixels in our case) to a two-dimensional Gaussian function. The third step is to link the localizations together from one frame to the next to create a particle trajectory which is as long as possible. These three steps combine to determine whether a particle can be successfully tracked. If a particle is not detected in every frame through the sequence, the trajectory will be truncated at the point where the particle was lost. MAP-SIM offers very high optical sectioning ability [4]. Because of this, the signal to background ratio (SBR) of the particles is higher, and the particles are thereby much easier to detect in the images. Our single particle tracking algorithm [16] is state of the art, however, in this particular case, it is unable to detect dim, faster moving particles in the widefield data consistently enough to be able to build a trajectory longer than a few frames. However, using MAP-SIM, we were able to successfully track these particles.

In this experiment, trajectories of single LAMP1-GFP particles (lysosomes, endosomes, or other vesicles containing LAMP1-GFP), were obtained by a SPT algorithm implemented in MATLAB [16]. Briefly, the intensity average of the reconstructed WF or MAP-SIM image stack was subtracted from each individual image within the stack to reduce sCMOS camera-induced fixed pattern noise and for feature enhancement. This is shown in Fig. S2(a). Single particle trajectories were determined from the processed data sets by selecting the initial starting particle coordinates by hand, Gaussian fitting of the imaged particle, and building trajectories from coordinates based on determining the probability of finding a diffusing particle in two dimensions at a given distance from its starting point after a given time [16]. The particles were tracked for at least 12 up to a maximum of 132 time steps of 250 ms each. Fig. S2(b) shows an example trajectory of a tracked particle. After this process of building uninterrupted trajectories, the mean-squared displacement (MSD), was calculated. The MSD is a measure of the average speed a particle travels and is calculated for each time difference *∆t* in the track. The MSD plot was computed up to *n∆t* < 1/4 of the total number of acquired time frames, where *n* is the number of available displacements of a given duration *n∆t* in the track record [12,16].

We tracked 60 LAMP1-GFP particles in the MAP-SIM image sequence taken from Fig. 4 of the main paper. Figure S3(a) depicts the corresponding MSD plots and Fig. S3(b) shows a histogram of the number of particles with a given hop speed computed from the average distance traveled during the shortest lag time, *∆t* = 250 ms.

We attempted to track the same particles in the WF image sequence using the same initial particle coordinates and tracking algorithm. We kept all algorithm settings the same between the two data sets except for the size of the point spread function. Of the 60 particles tracked in MAP-SIM, 39 or 65% were successfully tracked in the WF data. Figure S3(d) depicts the corresponding MSD plots obtained from the WF single particle trajectories and Fig. S3(e) shows a histogram of the number of particles with a given hop speed. Comparing the MAP-SIM MSD plot, Fig. S3(a), to the WF MSD plot, Fig. S3(d), it is evident that fast moving LAMP1-GFP particles, represented by steeper MSD curves, cannot be tracked as successfully in the WF data. As noted above, MAP-SIM increased the SBR of the particles we attempted to track. This is shown in Figs. S3(c) and S3(f). To evaluate the SBR of the particles, we evaluated a region of interest (ROI) around each particle (9x9 pixels). We calculated the local SBR as the ratio between the maximum (average of the 5% of the highest pixel values) and minimum (average of the 5% of the lowest pixel values) in the region. To analyze single particle tracking (SPT) experiments, we used custom routines [16,17] in MATLAB using DIPimage [18].


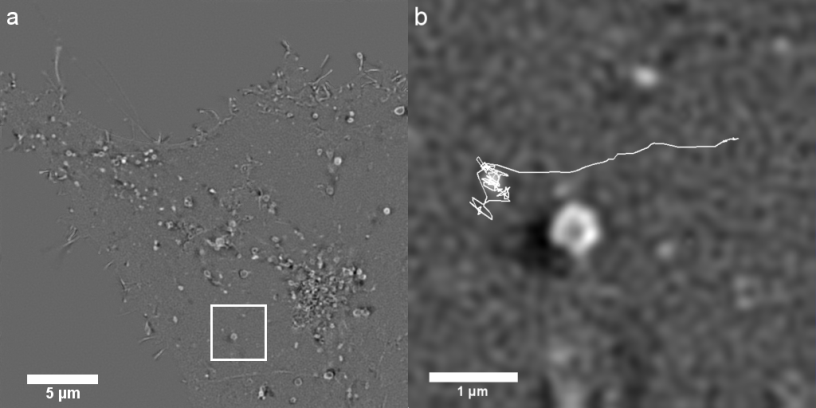


Fig. S2. Single particle tracking. (a) MAP-SIM image from Fig. 2(c) after subtraction of the average of the stack of images. (b) LAMP1-GFP particle trajectory (132 frames) within the boxed region in (a). The particle exhibits confined diffusion followed by directed movement.


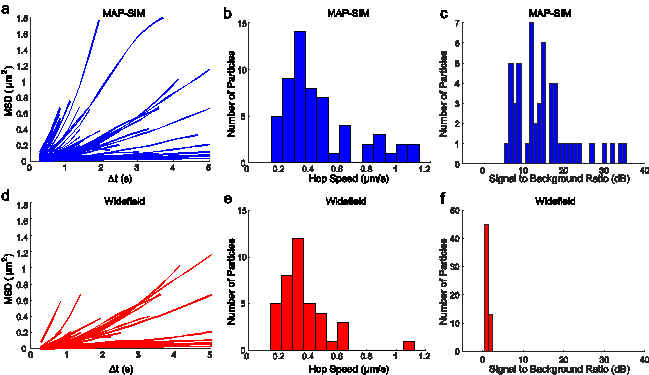


Fig. S3. MSD plot of (a) 60 LAMP1-GFP particle trajectories obtained from a MAP-SIM image sequence and (d) 39 LAMP1-GFP particle trajectories obtained from WF image sequence. (b) and (e) histograms of particle hop speeds computed for the average distance traveled during the time lag ∆t = 250 ms. (c) and (f) histograms of the signal to background ratio calculated for each particle that was tracked in the MAP-SIM (c) and WF (f) data.

**3. Changing the exposure time affects the resolution achieved in SIM experiments**

As noted in the main paper, the FLCOS microdisplay (and vendor-supplied microdisplay-timing program) we used can display an illumination pattern and switch to the next pattern in the sequence in 1.14 ms, allowing unprocessed SIM images to be acquired at rates of approximately 875 Hz. An exposure time of about 1 ms would be needed to achieve this imaging rate. However, such rapid imaging is not useful if the reconstructed SIM images are of poor quality, for example if they suffer from low signal to noise ratios (SNR). Specifying the fastest possible acquisition rate is thus inadequate without consideration of the resolution and SNR of the results.

Figure S4 demonstrates the effect of varying the camera exposure time on PSD_ca_ and thus on the spatial resolution. We imaged LAMP1-GFP cells and varied the camera exposure time from 10 ms to 100 ms per SIM sub-image. Low signal to noise ratios (in this case due to a short exposure time) causes a loss of fine details and therefore a reduction in effective resolution as estimated by our Fourier domain method [17]. These findings are not surprising, but the relationship between SNR (and therefore exposure times and usable imaging rates) and image resolution is typically not discussed in the SIM literature. This effect sometimes leads to very high noise and lower effective resolution when trying to push SIM imaging rates.


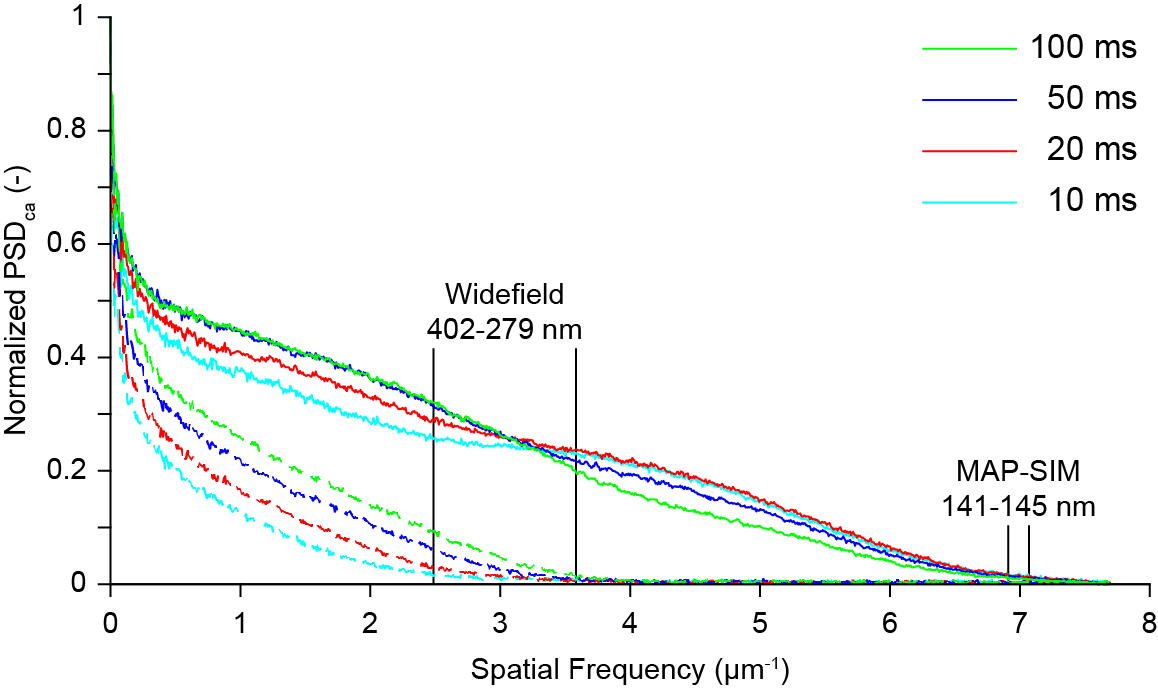


Fig. S4. Normalized circular average power spectral density (PSD_ca_) measured as a function of exposure time for WF and MAP-SIM.

**4. Comparison of MAP-SIM and spinning disk confocal microscopy**

We also used LAMP1-GFP cells to compare MAP-SIM with a spinning disk confocal microscope
(Andor Revolution system with Olympus IX81 microscope and UPLSAPO 100×/1.40 NA oil immersion objective). The results indicate a resolution of 342 nm for spinning disk confocal, 279 nm for WF, 286 nm for OS-SIM, and 145 nm for MAP-SIM.


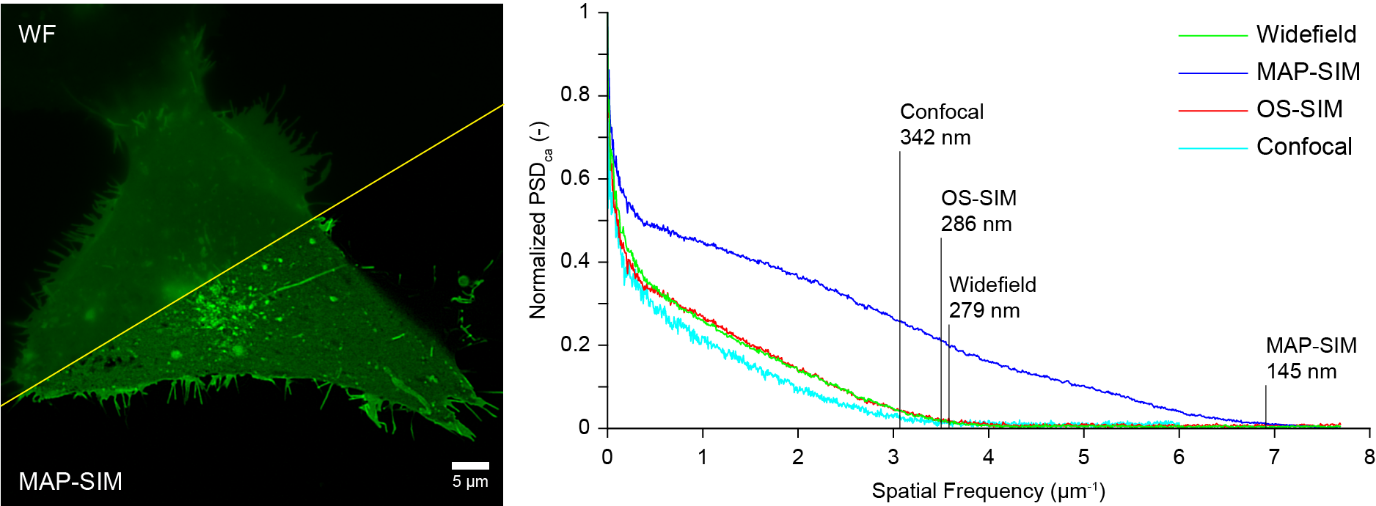


Fig S5. (at left) U2-OS cells expressing LAMP1-GFP, WF and MAP-SIM. (at right) PSD measurements for WF, OS-SIM, MAP-SIM, and spinning disk confocal.

**References**

1. R. Heintzmann, "Structured illumination methods," in *Handbook of Biological Confocal Microscopy*, J. B. Pawley, ed., 3rd ed. (Springer, 2006), pp. 265–279.

2. P. Křížek, I. Raška, and G. M. Hagen, "Flexible structured illumination microscope with a programmable illumination array," Opt. Express **20**, 24585 (2012).

3. M. A. A. Neil, R. Juškaitis, and T. Wilson, "Method of obtaining optical sectioning by using structured light in a conventional microscope," Opt. Lett. **22**, 1905–1907 (1997).

4. T. Lukeš, P. Křížek, Z. Švindrych, J. Benda, M. Ovesný, K. Fliegel, M. Klíma, and G. M. G. M. Hagen, "Three-dimensional super-resolution structured illumination microscopy with maximum a posteriori probability image estimation," Opt. Express **22**, 29805–17 (2014).

5. J. W. Goodman, *Introduction to Fourier Optics*, 2nd ed. (McGraw-HIll Int., 1996).

6. F. Orieux, E. Sepulveda, V. Loriette, B. Dubertret, and J.-C. Olivo-Marin, "Bayesian estimation for optimized structured illumination microscopy.," IEEE Trans. Image Process. **21**, 601–14 (2012).

7. P. J. Verveer and T. M. Jovin, "Efficient superresolution restoration algorithms using maximum a posteriori estimations with application to fluorescence microscopy," J. Opt. Soc. Am. A 1696–1706 (1997).

8. P. J. Verveer, M. J. Gemkow, and T. M. Jovin, "A comparison of image restoration approaches applied to three-dimensional confocal and wide-field fluorescence microscopy," J. Microsc. **193**, 50–61 (1999).

9. B. J. Vermolen, Y. Garini, and I. T. Young, "3D restoration with multiple images acquired by a modified conventional microscope," Microsc. Res. Tech. **64**, 113–125 (2004).

10. T. Lukeš, G. M. G. M. Hagen, P. Křížek, Z. Švindrych, K. Fliegel, M. Klíma, P. Krížek, Z. Švindrych, K. Fliegel, and M. Klíma, "Comparison of image reconstruction methods for structured illumination microscopy," Proc. SPIE **9129**, 91293J (2014).

11. S. Chaudhuri, *Super-Resolution Imaging* (CRC Press, 2011).

12. M. J. Saxton and K. Jacobson, "Single-particle tracking: applications to membrane dynamics.," Annu. Rev. Biophys. Biomol. Struct. **26**, 373–99 (1997).

13. K. Suzuki, K. Ritchie, E. Kajikawa, T. Fujiwara, and A. Kusumi, "Rapid hop diffusion of a G-protein-coupled receptor in the plasma membrane as revealed by single-molecule techniques," Biophyscial J. **88**, 3659–3680 (2005).

14. A. Kusumi, Y. Sako, and M. Yamamoto, "Confined lateral diffusion of membrane receptors as studied by single particle tracking (nanovid microscopy). Effects of calcium-induced differentiation in cultured epithelial cells," Biophys. J. **65**, 2021–2040 (1993).

15. N. Chenouard, I. Smal, F. de Chaumont, M. Maška, I. F. Sbalzarini, Y. Gong, J. Cardinale, C. Carthel, S. Coraluppi, M. Winter, A. R. Cohen, W. J. Godinez, K. Rohr, Y. Kalaidzidis, L. Liang, J. Duncan, H. Shen, Y. Xu, K. E. G. Magnusson, J. Jaldén, H. M. Blau, P. Paul-Gilloteaux, P. Roudot, C. Kervrann, F. Waharte, J.-Y. Tinevez, S. L. Shorte, J. Willemse, K. Celler, G. P. van Wezel, H.-W. Dan, Y.-S. Tsai, C. Ortiz de Solórzano, J.-C. Olivo-Marin, and E. Meijering, "Objective comparison of particle tracking methods.," Nat. Methods **11**, 281–9 (2014).

16. N. L. Andrews, K. A. Lidke, J. R. Pfeiffer, A. R. Burns, B. S. Wilson, J. M. Oliver, and D. S. Lidke, "Actin restricts FcepsilonRI diffusion and facilitates antigen-induced receptor immobilization," Nat Cell Biol **10**, 955–963 (2008).

17. J. Pospíšil, K. Fliegel, and M. Klíma, "Assessing resolution in live cell structured illumination microscopy," in *Proceedings of SPIE - The International Society for Optical Engineering*, P. Páta and K. Fliegel, eds. (SPIE, 2017), Vol. 10603, p. 39.
