# Supplementary material for: Imaging tissues and cells beyond the diffraction limit with structured illumination microscopy and Bayesian image reconstruction
Source: Gigascience. 2018 Aug 23;8(1):giy126. doi: 10.1093/gigascience/giy126 (PMC6325271; doi:10.1093/gigascience/giy126)
Supplement: Response_to_Reviewer_Comments_Original_Submission.pdf [file giy126_response_to_reviewer_comments_original_submission.pdf]

Author's Response To Reviewer Comments

Close

Dear Editor:

We would like to resubmit our manuscript entitled "Imaging tissues and cells beyond the diffraction limit with structured illumination microscopy and Bayesian image reconstruction" for consideration in GigaScience as a data note.

We would like to thank the referees for their very positive reviews of the paper. Reviewer 1 had a minor point of concern:

"One minor point the authors may want to address is the difference between coherent interference illumination and the incoherent pattern illumination employed in the manuscript. It seems that interference illumination can generate a sin pattern with highest contrast at cutoff frequency while incoherent pattern illumination may suffer from the gradually cutoff of the incoherent OTF."

We thank the reviewer for this comment. The reviewer is correct about this, and we have written some comments and added a new reference in the discussion which should clear up this matter. The modified paragraph now reads:

"There are several other advantages to the use incoherent illumination in SIM, including removing the need for a pupil plane mask to block unwanted diffraction orders. Also, incoherent imaging of a microdisplay for pattern formation means that the pattern spatial frequency in the sample plane does not depend on the wavelength of the light which is used. On the other hand, in incoherent illumination SIM such as we used here, the contrast of the illumination pattern decreases with increasing spatial frequency according to the incoherent optical transfer function [53]. In coherent illumination SIM [8,9,11], the coherent optical transfer function applies [53], and so the pattern contrast does not decrease with increasing spatial frequency. This means that coherent illumination SIM can more efficiently mix high resolution information from outside the frequency limit into the detection passband of the microscope, thereby potentially achieving better resolution than what we achieved in this work. We achieved a lateral resolution enhancement factor of ~1.8 (Fig. 2), whereas a factor of 2.0 is expected for coherent illumination SIM."

In addition to this, we have fixed a few typographical errors.

We hope that the paper will now be acceptable for publication.

Sincerely,

Guy M. Hagen

Close
