# Supplementary material for: Imaging tissues and cells beyond the diffraction limit with structured illumination microscopy and Bayesian image reconstruction
Source: Gigascience. 2018 Aug 23;8(1):giy126. doi: 10.1093/gigascience/giy126 (PMC6325271; doi:10.1093/gigascience/giy126)
Supplement: Reviewer_1_Report_(Original_Submission) -- Guoan Zheng — 8/16/2018 Reviewed [file giy126_reviewer_1_report_(original_submission).pdf]

Reviewer Report

Title: Imaging tissues and cells beyond the diffraction limit with structured illumination microscopy and Bayesian image reconstruction

Version: Original Submission **Date:** 8/16/2018

Reviewer name: Guoan Zheng

Reviewer Comments to Author:

This manuscript provides the raw and analyzed data for structured illumination microscopy. The authors discuss different processing methods and provide the corresponding open-source codes. The SIM data and the processing codes are highly desired for the microscopy imaging community. In particular, the high quality raw data provided by this manuscript will help researchers to develop new methods for SIM. One minor point the authors may want to address is the difference between coherent interference illumination and the incoherent pattern illumination employed in the manuscript. It seems that interference illumination can generate a sin pattern with highest contrast at cutoff frequency while incoherent pattern illumination may suffer from the gradually cutoff of the incoherent OTF.

Level of Interest

Please indicate how interesting you found the manuscript: Choose an item.

Quality of Written English

Please indicate the quality of language in the manuscript: Choose an item.

Declaration of Competing Interests

Please complete a declaration of competing interests, considering the following questions:

- Have you in the past five years received reimbursements, fees, funding, or salary from an organisation that may in any way gain or lose financially from the publication of this manuscript, either now or in the future?
- Do you hold any stocks or shares in an organisation that may in any way gain or lose financially from the publication of this manuscript, either now or in the future?
- Do you hold or are you currently applying for any patents relating to the content of the manuscript?
- Have you received reimbursements, fees, funding, or salary from an organization that holds or has applied for patents relating to the content of the manuscript?
- Do you have any other financial competing interests?

- Do you have any non-financial competing interests in relation to this paper?

If you can answer no to all of the above, write 'I declare that I have no competing interests' below. If your reply is yes to any, please give details below.

I declare that I have no competing interests

I agree to the open peer review policy of the journal. I understand that my name will be included on my report to the authors and, if the manuscript is accepted for publication, my named report including any attachments I upload will be posted on the website along with the authors' responses. I agree for my report to be made available under an Open Access Creative Commons CC-BY license (http://creativecommons.org/licenses/by/4.0/). I understand that any comments which I do not wish to be included in my named report can be included as confidential comments to the editors, which will not be published.

I agree to the open peer review policy of the journal

To further support our reviewers, we have joined with Publons, where you can gain additional credit to further highlight your hard work (see: https://publons.com/journal/530/gigascience). On publication of this paper, your review will be automatically added to Publons, you can then choose whether or not to claim your Publons credit. I understand this statement.

Yes
